# The Use of Low-Profile Angular-Stability Plates in a “Nutcracker” Tarsal Navicular Fracture Combined with a Cuboid Fracture: ORIF Experience

**DOI:** 10.3390/jfmk6040099

**Published:** 2021-12-06

**Authors:** Fabrizio Quattrini, Corrado Ciatti, Serena Gattoni, Calogero Puma Pagliarello, Francesco Ceccarelli, Pietro Maniscalco

**Affiliations:** 1 Orthopaedic and Traumatology Department, Ospedale Guglielmo da Saliceto, 29121 Piacenza, Italy; fabrizioquattrini@yahoo.it (F.Q.); dadociatti@icloud.com (C.C.); serenagattoni@gmail.com (S.G.); C.pumapagliarello@ausl.pc.it (C.P.P.); 2 Department of Medicine and Surgery, Orthopedic Clinic, University Hospital of Parma, 43100 Parma, Italy; francesco.ceccarelli@unipr.it

**Keywords:** navicular, cuboid, nutcracker fracture, midfoot, midtarsal joint

## Abstract

Background: Clear recommendations about the optimal treatment of traumatic tarsal navicular fractures are still very debated in the literature, and this is due to several factors: navicular fractures are rare and often misdiagnosed injuries, they are frequently associated with other fractures or a dislocation of the midfoot, and the current knowledge is based on few papers mainly considering a limited number of cases and dealing with different therapeutic approaches. The treatment of navicular body fractures is controversial and burdened by a high incidence of complications; in particular, Sangeorzan type III comminuted fractures represent a real challenge for the orthopedic surgeon. An accurate preoperative planning, a scrupulous surgical technique aimed at restoring volume and bony anatomy, and the use of low-profile angular-stability plates can lead to optimal clinical and functional results, decreasing the chances of arthritic evolution of mid-foot joints.

## 1. Introduction

Navicular fractures are extremely rare events. As a matter of fact, midfoot injuries represent about 5% of all foot traumas, and those affecting the Chopart joint are less than 20% of them [1]; therefore, less than 1% of all foot injuries are navicular fractures [2,3]. The diagnosis of navicular fractures has been described as “sometimes obvious, frequently difficult, and occasionally elusive”, and the entire scientific community agrees on the complexity of the diagnostic process [4]. Navicular body fractures are generally the result of a direct axial load secondary to a fall or of the impact of an indirect force. The pathomechanical description of these fractures includes the impact of the cuneiforms into the navicular during a longitudinal shear force compression, a lateromedial compression of the cuneiforms into the navicular, forcing it against the talar head with plantar-flexed ankle, and navicular compression into the talar head with dorsiflexion of the midfoot and eversion of the hindfoot [5,6].

The Sangeorzan’s classification divides navicular body fractures in three types, based on the plane of fracture and the degree of comminution: type 1 presents a dorsal fragment, type 2 a dorsomedial fragment, and type 3 is characterized by central comminution [7]. More recently, Schmid et al. classified navicular fractures according to the degree of talonavicular joint involvement in type 1 if they present a two-part fracture of the navicular body, type 2 in case of comminution, and type 3 if they are associated with talonavicular joint dislocation and/or talar head fracture [8].

The navicular bone lies in the medial longitudinal arch of the midfoot, part of the transverse tarsal joint, and it is articulated both with the hindfoot and with the forefoot. The talonavicular is the most mobile joint of the midfoot and, in association with the subtalar joint, it forms the “acetabulum pedis” [9]. This complex influences the mobility of the entire foot, particularly in pronation and supination. From a biomechanical point of view, the talocalcaneonavicular joint is responsible for the efficiency of gait [4,10]: when the hindfoot is everted, the talonavicular and calcaneonavicular joints have parallel axes, allowing flexion movements; on the other hand, when the hindfoot is not everted, these joints allow little motions inside the midfoot, forming a rigid lever arm, useful for the propulsive phase of gait [11,12]. Considering this key role in the medial longitudinal arch, fractures (or avascular necrosis) of the navicular bone can lead to arch collapse, painful flatfoot deformity, and progressive valgus deformity of the subtalar joint [12].

The navicular bone is vascularized by branches of both the dorsalis pedis artery on its dorsal path and the tibialis posterior via the medial plantar artery on its plantar and medial aspects [13], but nutrient arteries come from dorsal aspects of the bone [14]. The central part of the navicular bone is relatively avascular, and cadaveric studies demonstrated that 12% of specimens present a central avascular region; this peculiar vascularization increases the risk of delayed healing, avascular necrosis, and nonunion of body navicular fractures [15,16].

We describe a case of a comminuted fracture of the navicular body associated with a cuboid fracture, treated with open reduction and internal fixation, trying to highlight our main surgical steps to obtain a good anatomical reconstruction aimed at the full functional recovery of the patient.

## 2. Case Presentation

A healthy 66-year-old woman presented to the Emergency Room of our center (the Guglielmo da Saliceto Hospital, Piacenza, Italy), after a roadside accident with her bicycle. She reported acute severe pain in her left foot and a slight bruise on the ipsilateral knee. Physical examination revealed moderate foot edema along with severe pain at the midfoot level. No circulatory or neurological disorder about the knee was reported.

**Figure 1:**
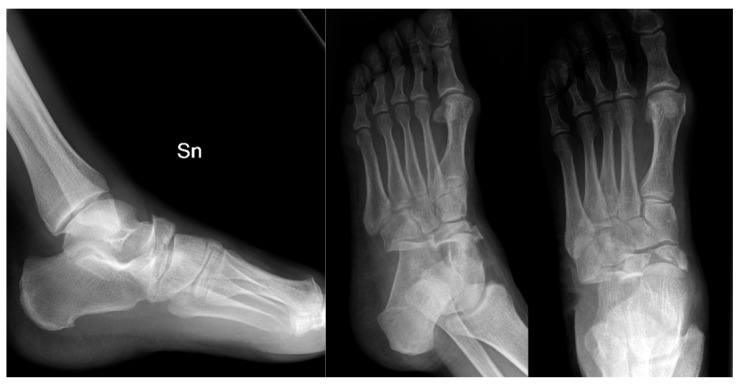
Preoperative X-rays.

Complete radiographic evaluation showed a complex fracture of the navicular body, an articular fracture of the cuboid bone at the calcaneocuboid joint, and subluxation of the midfoot (Figure 1). As noted by Sanders et al., when the medial navicular fragment is not damaged, the forefoot may displace laterally at the midtarsal joint with varus deviation [17].

**Figure 2:**
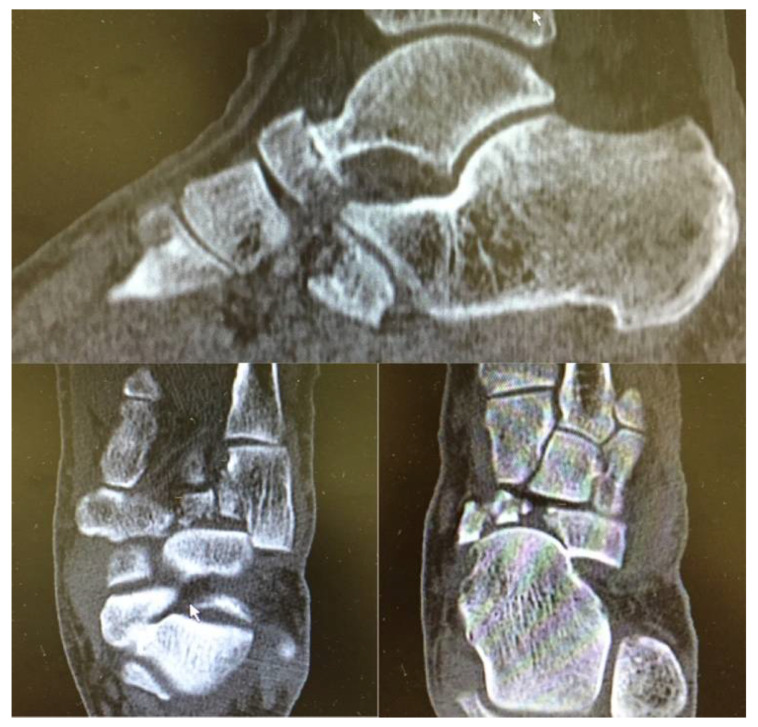
Preoperative CT.

CT scan confirmed a complex fracture of the navicular bone with comminution of the body, a cuboid fracture, and lateral subluxation of forefoot at the midtarsal joint (Figure 2). The pathogenetic mechanism of the injury consisted of a supination stress, classifiable as transnavicular according to Zwipp [18]; this process created a medial compression and a “nutcracker” mechanism, causing a comminuted fracture of the navicular body, a lateral distraction with a slight opening of the calcaneocuboid joint, and an impaction injury of the medial cuboid articular surface.

This navicular body fracture was classifiable as type 3 according to Sangeorzan’s classification and type 2 according to Schimd’s classification.

The foot was immobilized in a plaster cast, and surgery was scheduled for the next day. Preoperative clinical re-examination confirmed a moderate soft tissue edema compatible with the trauma; surgery was performed within 24 h from the event.

During the preoperative planning, we considered verifying the reducibility of the fracture with indirect maneuvers and the consequent possible stabilization with an external fixator and/or K-wires. In case of failure of these procedures, an open reduction and internal fixation (ORIF) were the mandatory option. This approach required a longitudinal dorsal access to the navicular bone, reduction of the fragments with possible addition of bone graft, synthesis with specific plate and screws; thereafter, a lateral access to expose the cuboid and lift the impacted area (even with the use of bone graft) and synthesis with another specific plate would be required.

## 3. Surgical Procedure

Surgery was performed with the patient in supine position and under an image intensifier’s guidance. A tourniquet was positioned at the distal part of the limb. After an anterior access between the extensor hallucis longus (E.H.L.) and the tibialis anterior (T.A.) tendons, we displayed the dorsal fragment of the navicular to apply traction on the longitudinal axis and restore its normal length, thus reducing the subluxation with longitudinal traction. An external fixator could be useful to maintain length and reduction in case of instability. We needed to fill the bone gap through a synthetic bone graft (NEOBONE^®^, CoorsTek, Golden, CO, USA, Hydroxyapatite synthetic bone substitute).

**Figure 3:**
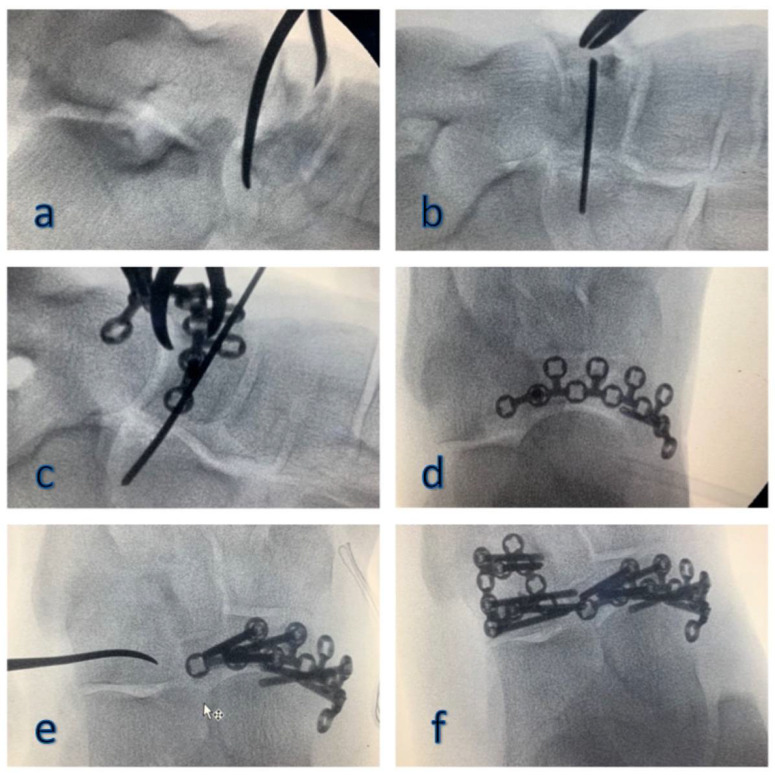
Intraoperative images: reduction (**a**), stabilization (**b**) and synthesis with plate and screws (**c**,**d**) of the navicular fracture; reduction and synthesis with plate and screws of the cuboid fracture, using the synthetic bone graft to fill the gap (**e**,**f**).

In order to reduce the navicular bone on the horizontal axis, a Weber forceps was anchored to the two major fragments involved, the medial plantar fragment and the dorsolateral margin (Figure 3a). Once we obtained the correct placement, we stabilized it with a K-wire (Figure 3b). Subsequently, we shaped a specific navicular plate (DePuy Synthes^®^, Warsaw, Indiana, variable-angle 2.4 navicular plate) which could perfectly adhere to our fragments; to finalize the synthesis, we extended the medial incision and implanted the plate along with all the necessary screws (Figure 3c,d).

Dorsolaterally approaching the cuboid, between the distal calcaneus and the base of the IV–V metatarsal, we opened a small bone window and lifted up the impaction fragment (Figure 3e); the gap was filled with synthetic bone and finally, using a specific plate (DePuy Synthes^®^, Warsaw, Indiana, variable-angle 2.4 cuboid plate) with the same characteristics of the one used before, we proceeded with the osteosynthesis (Figure 3f).

**Figure 4:**
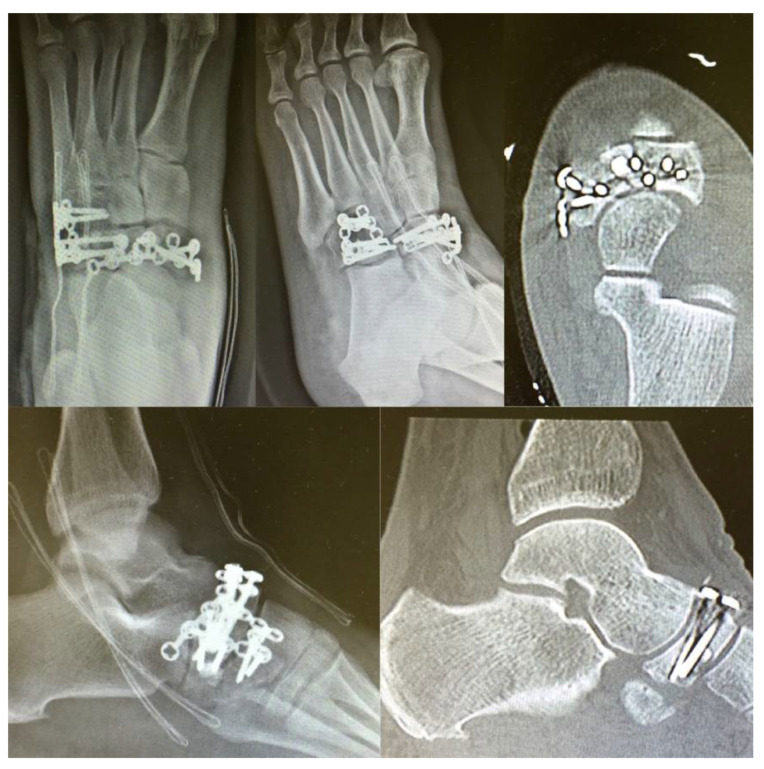
Postoperative X-rays imaging and CT scan.

At the end of the procedure, we checked the stability of fragments, confirming the good firmness of the synthesis and the absence of any subluxation. In conclusion, the foot was immobilized with a splint, and postoperative X-rays analysis and CT scan were executed (Figure 4).

The patient kept the splint for 2 weeks in order to maintain a neutral position and avoid the typical equinism tendency due to postoperative pain. We forbade weight bearing for the first 8 weeks; from weeks 8 to 12, we granted partial load, permitting full load only during pool rehabilitation; at 12 weeks, we permitted full weight bearing.

**Figure 5:**
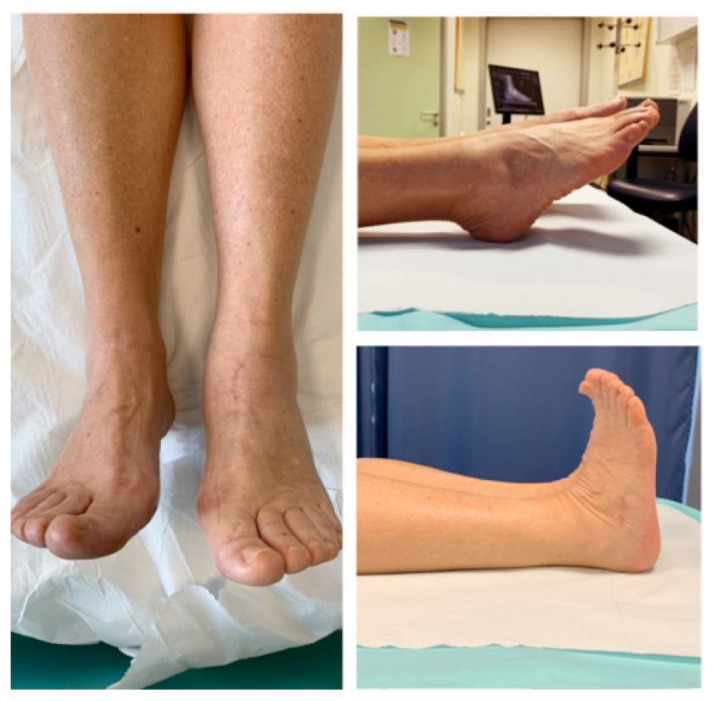
Follow-up after 4 months.

**Figure 6:**
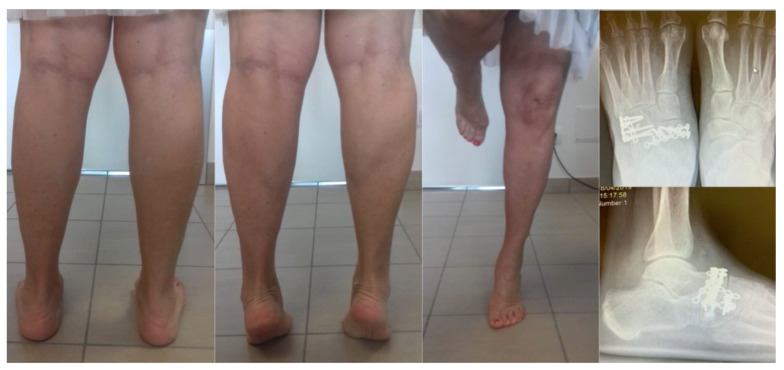
Twenty-four-month follow-up.

After 4 months from surgery, the patient attained the full range of motion and pronosupination, no pain, and normal walking phases (Figure 5), being able to walk on uneven ground and on tiptoe after 6 months (Figure 6). At 12 and 24 months of follow-up, she did not show any sign of arthritis.

## 4. Discussion

Specific midfoot plates present a particular anatomical conformation that guarantees a good coupling with bone shape, and a low profile allows avoiding damage to ligaments, soft tissues, and vessels, helping to preserve the blood supply to the navicular bone. Furthermore, plates can be contoured by surgeons considering the specific patient’s anatomy. A Variable-Angle Locking System creates a stronger construct and it is ideal for osteopenic bones, since it permits to share the load between plate and screw, increasing fixation stability. Moreover, the system improves the surrounding environment, accelerating bone healing, the rehabilitation period, and the patient’s return to previous mobility and function.

Comminuted fractures are particularly difficult to treat, and partial avascular necrosis, alone or associated with a secondary collapse, is frequent after a tarsal navicular fracture [7,19]. Moreover, these fractures increase the risk of early osteoarthritis, sometimes associated with the collapse of the plantar arch.

The arthrodesis of the talonavicular joint, combined or not with a correction of the deformity, can be performed to treat pain; however, this is at the expense of the hindfoot range of motion [20]. The fusion of the talonavicular joint severely restricts the motion of the hindfoot to approximately 2% of the preoperative values, whereas calcaneonavicular arthrodesis leads to 67% of the range of motion in the talonavicular joint compared to the preoperative values [20]. Anatomical reconstruction with restoration of volumes of the navicular and cuboid bones can lead to optimal functional recovery and prevent early degenerative evolution or deformity.

A CT scan is necessary in case of Chopart lesions, both for apparently simple cases that may be undiagnosed or underestimated [1] and for more complex cases, as this exam could allow a better understanding of the fracture pattern, the elaboration of a precise surgical plan, and the choice of the adequate fixation device. In particular, CT scan reconstructions allow studying fracture morphology and fragments size, deciding where exactly to point clamps to obtain bone volume reconstruction, and understanding if bone augmentation is necessary.

The surgical correction of the length and shape of the longitudinal arch is important but could be technically challenging; the restoration of the medial and lateral columns has a strong correlation with the functional outcome [3].

The reconstitution of navicular volumes, primarily the plantar fragment, is the keystone of navicular fractures treatment to avoid the failure of the medial longitudinal arch of the foot.

Prathapamchandra T. al. confirmed that the reduction of the plantar fragment seen pre-operatively on 3D CT reconstructions is an important method aiding anatomical reconstruction and fixation [14].

A bone graft can facilitate anatomical restoration, especially of medial column length, and fracture healing due to its osteoconductive and osteoinductive effects [21], improving the clinical and functional outcome of patients without lengthening hospitalization or recovery time [22].

The navicular bone is poorly vascularized; Torg et al. [23] in microangiographic studies showed that its lateral and medial thirds are adequately supplied by blood vessels, while the central third is almost avascular [9]; this probably explains the increased rate of non-union and avascular necrosis seen following fractures of this bone [17]. The blood supply of the navicular derives, dorsally, from a branch of the dorsalis pedis artery and plantarly, from a branch of the medial plantar artery; the medial tubercle receives blood supply from an anastomosis between the latter two. A rich anastomosis exists around the circumference of the navicular and a paucity of vessels supplying the central third in adults [17]. Van Langelan et al. showed that the central third of the navicular bone has an area of relative avascularity, while the medial and lateral thirds are well vascularized [24]. On the other hand, in another study, McKeon et al. reported that 58.8% of their analyzed patient cohort presented a good vascularization of the navicular bone, without avascular zones; only in 11.8% of the cases there was an avascular region in the dorsal, central third of the navicular; this area is the usual location of many stress fractures. Consequentially, these bones with a poor vascular supply are more prone to develop a navicular stress fracture [15].

Tissue respect during surgery and locking plate positioning reduce the risk of vascular injury; in addition, the possibility of slipping the plate under the soft tissues without periosteal stripping helps to achieve this goal [25]. In addition, surgeons should care not to strip the periosteum or the joint capsule from any small pieces. If a fragment is connected to the joint capsule, then the best solution is to flip it, so as not to disrupt its soft-tissue attachments; once the joint is reconstructed, this “trap door” piece can be reduced and fixed.

Compared to the traditional unlocked plates that were adapted, there are currently specific plates that can also be shaped in situ and allow the implantation of screws with angular stability.

In the postoperative period, a temporary immobilization with a neutral splint which helps avoiding the equinus position could be advisable. In fact, in the literature, there are many studies reporting that a gastro-soleus contracture may coexist in patients with a navicular fracture, and this leads to forefoot overload and deformity, although increasing rates of non-union, post-traumatic arthritis, and ongoing pain through this association have not yet been shown to be causal [26,27,28,29].

The use of the Silverskiold test intraoperatively could be useful to test the gastrocnemius tightness, and in the literature, some authors suggest to use the Strayer procedure during surgery to mitigate it in selected cases [26].

## 5. Conclusions

Cases like the one reported are fortunately rare but with potentially serious functional repercussions. For a long time, it was thought that immediate or delayed arthrodesis was the only solution. Actually, an accurate radiological study, a careful preoperative planning, and a meticulous surgical technique can lead to better results. Specific surgical instruments available today, plates in particular, allow us to perform accurate anatomical reconstructions with excellent stability, minimizing the risk of tissue suffering and the incidence of complications. Even in particular and complex cases, such as the one we have presented, today we can obtain excellent clinical and functional results.
